# Alcohol consumption among university students: a Sino-German comparison demonstrates a much lower consumption of alcohol in Chinese students

**DOI:** 10.1186/s41043-016-0062-0

**Published:** 2016-08-11

**Authors:** Janet Junqing Chu, Heiko J. Jahn, Mobarak Hossain Khan, Alexander Kraemer

**Affiliations:** 1 Department of Public Health Medicine, School of Public Health, Bielefeld University, Universitaetsstr. 25, Bielefeld, 33615 Germany; 2 Department of Public Health, College of Applied Medical Sciences, King Faisal University, Hofuf, Saudi Arabia

**Keywords:** Alcohol consumption, Students’ health, University students, Chinese students, German students

## Abstract

**Background:**

Alcohol use is reported in university students with discrepancy between countries. The study objectives were to assess prevalence and associated factors of alcohol consumption among university students in Germany and China.

**Methods:**

Data used were from 1853 Chinese and 3306 German university students. Alcohol consumption frequency was measured by a question “How often did you drink alcohol in the last three months?” with six possible responses, which were later collapsed into three categories of “At least once a week”, “Less than once a week” and “Never”. Problem drinking was measured by the CAGE test and defined as a CAGE score of two or more (four as the maximum). Simple and multivariable logistic regressions were used for association analyses.

**Results:**

German students reported more often “At least once a week” drinking (59.8 vs. 9.0 %). Among Germans, women drank less often “At least once a week” (OR = 0.40, 0.30–0.53). Among Chinese, a higher BMI was associated with drinking “At least once a week” (OR = 1.09, 1.02–1.18). Age revealed a positive association with “At least once a week” drinking in Chinese (1.33, 1.21–1.46) but a negative association in Germans (OR = 0.97, 0.94–0.99). Having a father with high educational level was positively related to “At least once a week” drinking in both countries (OR = 4.25, 2.67–6.78 for Chinese; OR = 1.32, 1.01–1.72 for Germans). Doing less than once a week physical exercise was negatively associated with “At least once a week” drinking in Chinese and German students (OR = 0.27, 0.15–0.48 for Chinese; OR = 0.69, 0.49–0.96 for Germans). Among the German students, 20.3 % reported problem drinking. Being a female (OR = 0.32, 0.26–0.40) and performing less than once a week physical activity (OR = 0.73, 0.56–0.95) were negatively associated with problem drinking, while having a father with high educational level (OR = 1.32, 1.09–1.60) and experiencing higher level of perceived stress (OR = 1.08, 1.04–1.13) were positively related to problem drinking.

**Conclusions:**

Country-specific strategies for reducing alcohol consumption, e.g. educational awareness programmes of alcohol use on Chinese campuses and alcohol prevention schemes among German youth before entering university, are sensible.

## Background

University students are at a specific stage to experience more freedom in making personal choices about their health behaviours than earlier or later in life [1–3]. Some forms of risky behaviours such as alcohol consumption peak in this age group [1–3]. Alcohol use is broadly reported among university students [2, 4, 5]. They seem to consume more alcohol than their counterparts in the general population [5, 6]. College students in many countries are at an elevated risk for problem drinking [3]. Alcohol places a significant burden on human life [7–9]. It can cause 60 different kinds of diseases and conditions, including injuries and mental and behavioural disorders [7, 10]. At the same time, alcohol increases the risk of a wide range of social harms in the student populations. Studies reported the connection between alcohol use and missing class [2, 3, 10], impaired academic achievement [3, 6, 11], unsafe sex [12] and violence [2, 12] among university students. Understanding prevalence and associated factors of alcohol consumption in this population could open important venues for intervention. Based on previous research, the associated factors of alcohol consumption in university students include demographic attributes, such as gender and age [3, 10]; family socio-economic status, such as income and parent educational level [3, 7, 11]; and lifestyle-related factors, such as subjective health [11], physical activity [3, 13], nutrition awareness [14, 15] and perceived quality of life [16], social activity [10, 17] and study-related stress [3, 10, 13]. Ethnic group differences were found by different studies, e.g. it has been reported that Caucasians drank more than Asians [8, 18, 19]. Cultural differences were also reported, and in general, alcohol consumption was higher among students in Europe and North America than in Asia and Africa [5]. Along with the process of globalization and increasing international student exchange, it is of growing importance to conduct cross-country comparative studies in alcohol consumption prevalence and associated factors [8, 20]. Particularly interesting may be comparative studies between China and Germany in this regard due to the following reasons: Firstly, until recently, alcohol use-related problems were not common in China like in western countries. Alcohol use disorders are only beginning to emerge in Chinese society and at the university; therefore, scientific publications in this field are still scarce [20, 21]. Secondly, the academic exchange between China and Germany has increased considerably in the last decade. Students of Chinese origin have become the biggest foreign student group at German universities since 2010 [22]. Thirdly, to our knowledge, there is no study published yet on alcohol consumption pattern comparing Chinese and German university students.

Based on the gaps mentioned above, we conducted two surveys in China and Germany among university students (*N* = 5159) with the objectives to assess and compare (1) the prevalence of alcohol consumption and (2) associated factors with alcohol consumption among university students in Germany and China. Our findings can provide information for educators and policymakers in China and Germany to implement target-orientated interventions against alcohol abuse at universities in both countries. The results of this study may also add evidence to university administrators and public health educators elsewhere dealing with students from China and Germany.

## Methods

### Survey and questionnaire

The data used in the analyses were extracted from two health surveys. Firstly, a multicenter student health survey was administrated in 2006–2007 at 16 universities in North Rhine-Westphalia (NRW), the biggest state in Germany. Twelve of the 13 general universities in NRW were included (one university, which was linked with a specific insurance company, was excluded). To match the ratio of 3:1 of students from general and applied science universities in NRW, four from all 25 universities of applied sciences in NRW were selected randomly. It was planned to include at least 200 students at each participating site, and because variation in the size of the surveyed courses was expected, the study was planned to include between 200 and 400 students in each site. Initially, a balanced sample of students with respect to study duration and proportion of natural and social sciences was planned, since differences in health, perceived stress and health-related behaviours between students of natural sciences and social sciences in different study years have been reported [23, 24]. Initially, a balanced sample of students with respect to study duration and proportion of natural and social sciences was planned. Courses were selected partly randomly and partly by convenience within these predefined groups. Due to organizational difficulties and variation in response rates (which ranged from 69 to 100 % in different universities and was on average 88 %), this plan was not fully realized. The final German sample included 3306 respondents, and students of medicine and health sciences and educational sciences as well as sport sciences were overrepresented. Secondly, a Chinese student health survey was accomplished in 2010–2011. Two large comprehensive universities—Sun Yat-sen University (SYSU) in Guangzhou and Peking University (PKU) in Beijing (one from southern China and one from northern China) that recruit students from the whole country—were selected. The sample was planned to present the university structure in terms of student proportion in natural sciences and social sciences, as well as study year. Altogether, 1853 undergraduate students completed the questionnaire with approximately half the sample coming from each university. The response rate was above 90 % (average response rate 91 %) at both universities. Due to organizational difficulties, the sample provided more variation than originally planned, and students from health sciences were overrepresented.

In the two countries, all universities involved gave permission to conduct the survey. An ethical approval for the study was obtained from the Institutional Review Boards of Peking University (Reference No. IRB 00001052-10082). The students were asked to complete the survey questionnaires at the end of lectures in the lecture rooms. They were informed in writing that participation was voluntary and anonymous; they agreed to participate by completing and returning the questionnaire. No incentives were provided for participation in the survey. In both surveys, a self-administered pre-tested questionnaire that contained information concerning socio-economic and demographic information, lifestyle-related attributes and perceived stress scale was used.

### Variables

#### Dependent variables

Alcohol consumption was measured by two variables. The first variable was alcohol consumption frequency, and it was measured by the question “How often did you drink alcohol in the course of the last three months?” with six possible options namely “Many times per day”, “Every day”, “Many times per week”, “Once per week”, “Less frequent than once a week” and “Never”. Due to the relatively low consumption of the Chinese subsample, respondents’ answers for alcohol consumption frequency were recorded into three categories of “At least once a week”, “Less than once a week” and “Never” (reference category) for performing simple and multivariable multinomial logistic regression in students from two countries.

The second variable was problem drinking prevalence. For identifying problem drinking, the four-item form of the CAGE test was applied. The CAGE test consists of four dichotomous questions: “Have you ever felt you should CUT down on your drinking?”; “Have people ANNOYED you by criticizing your drinking?”; “Have you ever felt bad or GUILTY about your drinking?”; and “Have you ever had a drink first thing in the morning (as an ‘EYE opener’) to steady your nerves or get rid of a hangover?” [4]. Problem drinking is defined as a CAGE score of two or more (four as the maximum) [25]. Due to the low prevalence of problem drinking among the Chinese students, there were not enough cases to be used in follow-up analysis. Therefore, binary logistic regressions were only conducted among German students to identify associations between demo-social factors and problem drinking (“Problem drinking” vs. “No problem drinking”, “No problem drinking” as the reference category).

#### Independent variables

The demographic and socio-economic information (five variables) included gender, age, degree of father’s education (“at least college level” [“high”]/“lower than college level” [“low”]), income sufficiency (“sufficient”/“insufficient”) and having a partner (“yes”/“no”).

The lifestyle-related characteristics (seven variables) included subjective general health (“good”/“poor”), perceived quality of life (“good”/“poor”), importance of nutrition (“important”/“unimportant”), importance of good grades at university (“important”/“unimportant”), physical activity (“less than once a week”/“once to twice a week”/“at least three times a week”), body mass index (BMI, calculated from self-reported weight and height using the metric BMI formula: BMI = weight in kilogramme divided by the square of height in metres) and perceived stress. Perceived stress was assessed by Cohen’s Perceived Stress Scale (PSS) [26] that evaluates the extent to which participants appraised life situations over the past month. The scale yields a single score per respondent, with higher scores indicating higher levels of stress. Cronbach’s alpha of the PSS was 0.77 for the Chinese (PSS-14) and 0.47 for the German (PSS-4) subsamples.

### Statistical analysis

Frequency, chi-square test (categorical variables) and Mann-Whitney *U* test (continuous variables) were used to describe the sample and compare characteristics between Chinese and German students. Since the measurement of alcohol consumption frequency in our study is ordinal, as compared with nominal regressions, ordinal models can simplify interpretation; originally, ordinal regression was planned for assessing associations between alcohol consumption frequency and demo-social variables in our sample. However, the test of parallel lines by SPSS’s PLUM procedure indicated that our data violate the proportional odds assumption. According to Williams [27], the simplicity of ordinal models is achieved by imposing constraints on the relationship between regressors and the probabilities of the outcomes. In the case of violation of the proportional odds assumption, using ordinal models can lead to incorrect conclusions [27]. Therefore, we used multinomial logistic regression for association analyses between alcohol consumption frequency and independent variables. Although nominal models have more parameters to interpret, this complexity is transparent when probabilities are used for interpretation since software easily makes the computations [28]. Meanwhile, multinomial logistic regression has been used in previous studies for analysing associations between alcohol consumption and influencing factors among university students in different samples [29–32]. Our data met all assumptions for conducting multinomial regression. We merged the data of two surveys into one dataset for analyses. For identifying associations between alcohol consumption frequency, problem drinking and associated factors, we used simple multinomial and simple binary logistic regression, respectively. Finally we included gender, age and all variables that were statistically significantly associated with alcohol consumption frequency and problem drinking in simple regression into multivariable regression to assess adjusted associations of alcohol consumption and demographic, socio-economic and lifestyle-related factors. In multinomial regression, in order to assess the associations between alcohol consumption frequency and influencing factors in China and Germany, respectively, and compare potentially identical or different predictors of alcohol consumption between the two countries, at the same time, to avoid inclusion of extraneous variables (in the case of multivariable regression, interaction terms of variable “country” with every covariate in the model), we used the “split file – comparing groups” function to run logistic regression for the two subgroups (Chinese/German). The analysis was performed with IBM SPSS statistics 21.

## Results

### General characteristics and alcohol consumption by demo-social factors of the sample

The sample description is presented in Table 1. In general, the German sample was older, with higher BMI and more female students. In both countries, the majority of the students rated their general health as good. Chinese students reported a higher rate of sufficient income and a lower rate of good quality of life than their fellow German students. Alcohol consumption by demographic, socio-economic and lifestyle-related factors for each country is presented in Table 2. Among the German students, 20.3 % reported problem drinking.

**Table 1:** Characteristics of the sample by country

Variables		Percent		Chi-square test *p* value
		Chinese (*N* = 1853)	German (*N* = 3306)	
Gender	Male	52.1	47.3	0.001
	Female	47.9	52.7	
Having a partner	Yes	31.4	56.0	<0.001
	No	68.6	44.0	
Father’s education	High	45.8	52.8	<0.001
	Low	54.2	47.2	
Subjective general health	Good	89.2	87.0	0.025
	Poor	10.8	13.0	
Income sufficiency	Sufficient	81.2	58.5	<0.001
	Insufficient	18.8	41.5	
Nutrition importance	Important	89.3	91.8	0.003
	Unimportant	10.7	8.2	
Grade importance	Important	92.8	96.2	<0.001
	Unimportant	7.2	3.8	
Physical activity	≥3 times a week	22.7	41.3	<0.001
	1–2 times a week	48.8	36.2	
	Less than once a week	28.5	22.5	
Perceived life quality	Good	39.0	57.8	<0.001
	Poor	61.0	42.2	
Alcohol consumption	Never	50.4	9.8	<0.001
	Less than once a week	40.6	30.4	
	At least once a week	9.0	59.8	
		Median (quartile deviation)		Mann-Whitney *U* test *p* value
Age		21.0 (1.5)	23.0 (2.0)	<0.001
BMI		20.1 (1.6)	22.3 (1.9)	<0.001

**Table 2:** Alcohol consumption by demo-social factors

Variables		Percent							
		Chinese			German				
		Never	Less than once a week	At least once a week	Never	Less than once a week	At least once a week	No problem drinking	Problem drinking
Gender	Male	49.3	42.4	8.3	7.2	20.2	72.6	70.3	29.7
	Female	52.2	38.4	9.4	11.7	39.8	48.5	88.6	11.4
Having a partner	Yes	42.3	47.4	10.3	9.8	32.3	57.9	81.9	18.1
	No	54.5	37.6	7.9	9.4	28.0	62.6	77.4	22.6
Father’s education	High	48.2	38.8	13.0	8.8	29.1	62.1	78.1	21.9
	Low	53.3	42.1	4.6	10.6	32.0	57.4	82.2	17.8
Subjective general health	Good	51.3	39.9	8.8	9.3	30.3	60.4	80.2	19.8
	Poor	44.3	45.3	10.4	13.0	30.4	56.6	76.0	24.0
Income sufficiency	Sufficient	52.5	39.1	8.4	10.3	31.1	58.6	80.7	19.3
	Insufficient	42.2	46.7	11.1	8.4	29.7	61.9	79.0	21.0
Nutrition importance	Important	52.2	40.0	7.8	9.9	31.3	58.8	80.5	19.5
	Unimportant	41.1	44.3	14.6	8.7	19.5	71.8	71.0	29.0
Grade importance	Important	51.4	40.2	8.4	9.6	30.6	59.8	80.1	19.9
	Unimportant	41.7	44.1	14.2	11.7	25.8	62.5	68.6	31.4
Perceived life quality	Good	50.9	39.4	9.7	8.8	29.5	61.7	79.4	20.6
	Poor	50.4	41.3	8.3	11.0	31.8	57.2	80.0	20.0
Physical activity	≥3 times a week	42.6	44.4	13.0	8.3	28.5	63.2	76.0	24.0
	1–2 times a week	52.5	40.0	7.5	9.2	31.2	59.6	82.0	18.0
	Less than once a week	54.6	39.0	6.4	13.2	32.8	54.0	83.1	16.9
		Median (quartile deviation)							
Age		20.0 (1.5)	21.0 (2.0)	22.0 (2.0)	23.0 (2.0)	23.0 (2.0)	23.0 (1.5)	22.5 (2.0)	23.0 (2.0)
Perceived Stress Scale scores^a^		25.0 (5.0)	24.0 (5.5)	25.0 (4.0)	9.0 (2.0)	9.0 (1.5)	8.0 (1.5)	8.0 (1.5)	9.0 (2.0)
BMI		19.9 (1.5)	20.3 (1.6)	21.0 (1.7)	22.4 (2.4)	21.9 (1.8)	22.5 (1.8)	22.1 (1.9)	23.0 (1.7)

^a^Perceived stress scale scores range 0–56 for Chinese and 1–16 for German

### Bivariable associations between alcohol consumption frequency, problem drinking prevalence and demographic, socio-economic and lifestyle-related factors

The associations between alcohol consumption and related factors included in the investigation are presented in Table 3. High educational degree of the father and doing more often physical activity was associated with “At least once a week” alcohol consumption in both Chinese and German students. Gender difference in terms of males consuming more alcohol was only revealed in Germans. Having a partner was associated with more alcohol consumption in the Chinese but less problem drinking among the Germans. Age was positively associated with alcohol consumption among Chinese but negatively related to alcohol consumption frequency among Germans. In German students, perceived stress was positively related to problem drinking.

**Table 3:** Bivariable associations between alcohol consumption and demo-social factors: odds ratio (OR) and 95 % confidence interval (95 % CI)

Variables	OR (95 % CI)				
	Chinese		German		
	“Less than once a week” vs. “Never”^a^	“At least once a week” vs. “Never”^a^	“Less than once a week” vs. “Never”^a^	“At least once a week” vs. “Never”^a^	“Problem drinking” vs. “No problem drinking”^a^
Female vs. male^b^	0.86 (0.70–1.04)	1.07 (0.77–1.51)	1.21 (0.92–1.58)	0.41*** (0.32–0.53)	0.30*** (0.25–0.37)
Having a partner, yes vs. no^b^	1.63*** (1.31–2.01)	1.68** (1.17–2.41)	1.11 (0.86–1.44)	0.89 (0.70–1.13)	0.76** (0.64–0.91)
Father’s education, high vs. low^b^	1.02 (0.83–1.24)	3.12*** (2.13–4.56)	1.09 (0.84–1.41)	1.30* (1.02–1.66)	1.29** (1.08–1.54)
Subjective general health, good vs. poor^b^	0.76 (0.56–1.04)	0.73 (0.44–1.23)	1.40 (0.99–1.98)	1.50* (1.09–2.07)	0.78 (0.61–1.00)
Income, sufficient vs. insufficient^b^	0.67** (0.52–0.87)	0.61* (0.40–0.92)	0.85 (0.65–1.11)	0.77* (0.60–0.99)	0.90 (0.76–1.08)
Nutrition, important vs. unimportant^b^	0.71* (0.51–0.99)	0.42*** (0.26–0.68)	1.41 (0.85–2.34)	0.72 (0.46–1.12)	0.59*** (0.45–0.78)
Grade, important vs. unimportant^b^	0.74 (0.50–1.09)	0.48* (0.27–0.85)	1.44 (0.76–2.74)	1.16 (0.65–2.08)	0.54*** (0.36–0.81)
Perceived life quality, good vs. poor^b^	0.95 (0.77–1.16)	1.16 (0.82–1.64)	1.17 (0.91–1.51)	1.36* (1.07–1.72)	1.04 (0.87–1.23)
Physical activity, less than once a week vs. at least three times a week^b^	0.84 (0.68–1.05)	0.85** (0.38–0.87)	0.73* (0.55–0.97)	0.58*** (0.45–0.76)	0.65*** (0.51–0.82)
Physical activity, 1–2 times a week vs. at least three times a week^b^	0.90 (0.74–1.09)	0.68* (0.48–0.96)	1.14 (0.87–1.49)	1.09 (0.85–1.40)	0.70*** (0.57–0.85)
Age	1.14*** (1.08–1.19)	1.33*** (1.23–1.45)	0.97* (0.94–0.99)	0.96** (0.93–0.99)	1.01 (0.99–1.04)
Perceived stress scale scores	1.01 (0.99–1.02)	1.02 (0.99–1.04)	0.98 (0.93–1.03)	0.96 (0.92–1.01)	1.05** (1.02–1.09)
BMI	1.07*** (1,03–1.12)	1.15*** (1.09–1.23)	0.96* (0.92–0.99)	1.01 (0.97–1.04)	1.05*** (1.03–1.08)

Significance of Wald test: *p < 0.05; **p < 0.01; ***p < 0.001

^a^Reference category of the dependent variables

^b^Reference category of the independent variables

### Multivariable associations between alcohol consumption, demographic, socio-economic and lifestyle-related factors

Table 4 presents the statistically significant associations between alcohol consumption and demographic, socio-economic and lifestyle-related factors in multivariable logistic regressions. For both Chinese and Germans, having a father with high educational degree was positively associated with “At least once a week” alcohol consumption, while having sufficient income and doing less than once a week physical activity were negatively related to drinking at this level. Age showed a positive association with “At least once a week” drinking among the Chinese but a negative association among the Germans. In German students, problem drinking was associated with being a male, performing at least three times a week physical activity and experiencing higher level of perceived stress.

**Table 4:** Multivariable regression: odds ratio (OR) and 95 % confidence interval (95 % CI) for alcohol consumption

Variables	OR	95 % CI
“Less than once a week” vs. “Never”^a^ (Chinese^b^)		
Having a partner, yes vs. no^c^	1.47**	1.16–1.85
Income, sufficient vs. insufficient^c^	0.72*	0.54–0.96
Physical activity, less than once a week vs. at least three times a week^c^	0.55***	0.40–0.75
Physical activity, 1–2 times a week vs. at least three times a week^c^	0.70*	0.54–0.93
Age	1.12***	1.06–1.18
BMI	1.09***	1.04–1.13
“At least once a week” vs. “Never”^a^ (Chinese^b^)		
Father’s education, high vs. low^c^	4.25***	2.67–6.78
Income, sufficient vs. insufficient^c^	0.49**	0.29–0.82
Nutrition, important vs. unimportant^c^	0.49*	0.27–0.89
Physical activity, less than once a week vs. at least three times a week^c^	0.27***	0.15–0.48
Physical activity, 1–2 times a week vs. at least three times a week^c^	0.43***	0.27–0.69
Age	1.33***	1.21–1.46
BMI	1.09*	1.02–1.18
“Less than once a week” vs. “Never”^a^ (German^d^)		
Income, sufficient vs. insufficient^c^	0.74*	0.56–0.99
“At least once a week” vs. “Never”^a^ (German^d^)		
Female vs. male^c^	0.40***	0.30–0.53
Father’s education, high vs. low^c^	1.32*	1.01–1.72
Income, sufficient vs. insufficient^c^	0.67**	0.51–0.88
Physical activity, less than once a week vs. at least three times a week^c^	0.69*	0.49–0.96
Age	0.97*	0.94–0.99
“Problem drinking” vs. “No problem drinking”^a^ (German^e^)		
Female vs. male^c^	0.32***	0.26–0.40
Father’s education, high vs. low^c^	1.32**	1.09–1.60
Physical activity, less than once a week vs. at least three times a week^c^	0.73*	0.56–0.95
Perceived stress scale scores	1.08***	1.04–1.13

Significance of Wald test: *p < 0.05; **p < 0.01; ***p < 0.001

^a^Reference category of the dependent variables

^b^Nagelkerke *R* ^2^ = 0.13 (df = 24, *N* = 1571, *p* < 0.001)

^c^Reference category of the independent variables

^d^Nagelkerke *R* ^2^ = 0.09 (df = 24, *N* = 2954, *p* < 0.001)

^e^Nagelkerke *R* ^2^ = 0.10 (df = 10, *N* = 2907, *p* < 0.001)

## Discussion

### Main findings of this study

Our study investigated alcohol consumption and factors associated with it among 5159 university students in China and Germany. We found that the German students consumed more alcohol than the Chinese. Regarding factors associated with drinking, we found differences between the two subsamples, such as gender difference in terms of males consuming more alcohol revealed only among the Germans, while a positive association between alcohol consumption, BMI and paying less attention to nutrition was only found among the Chinese. We also found common associated factors between the two countries, such as a positive association between “At least once a week” alcohol use, doing at least three times a week physical activity and having a father with high educational degree. At the same time, we identified difference of the association between the same factor “age” and “At least once a week” drinking between the two groups, i.e. a positive association in Chinese and a negative association in Germans. Our analyses show that perceived stress is related to problem drinking, but not to occasional alcohol consumption.

### What is already known on this topic

We found that perceived stress was not associated with drinking at levels of once a week or less than once a week but positively associated with problem drinking. This is consistent with findings from previous studies that drinking as a coping strategy predicted problem drinking, not non-problem drinking [3, 10, 11]. Our finding that high educational degree of the father was associated with more alcohol consumption in both Chinese and German students is in agreement with previous studies that drinking was related to social economic status (SES). Those with low SES are less likely to drink alcohol [3, 7]. We found that more frequent physical activity was related to more alcohol use in both Chinese and German students, and this lends support to previous findings that sport was associated with social drinking [10] and increased alcohol consumption may serve as a strategy for coping with aversive social interactions [17]. Our findings of a positive association between drinking, BMI and paying less attention to nutrition among Chinese were in agreement with previous studies conducted among Asian students [14, 15]. Despite their relatively low BMI, many university students in China, Japan and Korea had a greater desire to be thinner; they were very strict with drinking and consumption of certain foods [14, 15].

### What this study adds

We found relatively low alcohol use among Chinese students compared to German students, and drinking was positively associated with age among the Chinese but negatively among the Germans. These findings may be explained to a certain extent by the following: Firstly, our Chinese sample was younger than their German counterparts (21 vs. 23 years). According to a nationwide study conducted in 2007 among 159,117 subjects aged 15 years and above in China, only 8.8 % of the current drinkers had had their first alcoholic drink before 18 years and the peak alcohol consumption age of this population was 45–59 years [21]. On the contrary, the reported onset age of drinking among Europeans was around 12 years, at 15–16 years many of them had already binge drinking experience [7, 12]. The peak alcohol consumption age in European young people was supposed to be 18–20 years, drinking decreased along with years afterwards [8, 11]. Secondly, the varied culture and social norms in the two countries may play roles in influencing students’ drinking behaviour. For example, the typical expression used in China for keeping a social network is “let’s have a meal together to talk about old times” [20], and in Germany, one may say “let’s have a drink together to catch up with one another” for the similar purpose. According to WHO, the yearly alcohol consumption per person in EU was higher than that in China (8.6 vs. 4.8 l in 2001) [9]. Thirdly, ethnic differences in drinking patterns were reported by many studies among university students. For instance, in a study conducted at a Canadian university, Li and Rosenblood found that Caucasian students drank more than Asians students [19]. Webb et al. reported that the non-drinker rate was lower in white than non-white among British students (6 vs. 58 %) [8]. Fourthly, the so-called Asian flush, may have impact on alcohol use. Under normal circumstances, the human body first breaks down alcohol by alcohol dehydrogenase (ADH) into acetaldehyde, which is then converted by aldehyde dehydrogenase (ALDH) into acetate. Goedde et al. reported that about 50 % of Japanese and Chinese are deficient in ALDH, and this deficiency was detected in almost none of the Caucasoid and Negroid examined so far [33]. Those with deficient ALDH respond to a mild dose of ethanol with marked adverse reactions, such as facial flushing, increase of heart rate, hot feeling in the stomach, palpitations, tachycardia and muscle weakness [34]. These responses are thought to discourage the use and abuse of alcohol in the population. A study which assessed the association of ALDH status with binge drinking among 328 college students shows that having an inactive ALDH was a protective factor for binge drinking [18].

We found no gender difference in drinking among the Chinese. On the one hand, it may be explained by the findings of Bewick et al. that a gender difference was only found in high-level drinkers not in low-level drinkers [11], as only 9 % of the Chinese students consumed alcohol above once per week level. On the other hand, alcohol use differences were found in different regions in the general population in China [20]. As the two Chinese participating universities are elite universities with the students enrolled from the whole country, there might be some difference in drinking pattern between our subjects and students of provincial universities that recruited mainly from local areas. For example, a study conducted in Nanning, Guangxi province, China, reported a gender difference [35]. In our study, the high rate (66 %) of only-children among the Chinese sample may also influence these results. According to Katz and Boswell, the gender gap was found reduced among only-children as compared to non-only children, i.e. with only-child girls behaving more like boys [36].

In China, alcohol consumption among university students seemed to be increasing in recent years. A study conducted in 2013 among 1279 participants from six universities in China shows that 43.3 % students were regular drinkers (at least once a week), 36.7 % were occasional drinkers (less than once a week) and males were more likely to be a regular drinker compared with females [32]. It has been reported that among the Chinese students studying in the USA, those who were more socially affiliated with American culture were more likely to be regular drinkers [37]. While among the Chinese students in China, those who had western cultural orientation were more likely to be a regular drinker compared with those who had traditional Chinese orientation [32]. Due to the ALDH deficiency, in general, problem drinking has more potential harm for the Chinese students compared with their fellow European students. It is of importance to observe the drinking patterns of Chinese students studying in Germany in longitudinal studies in the future.

In Germany, problem drinking appeared to be growing in the last decade. Based on the Federal Centre for Health Education [38], the recent 30-day prevalence of alcohol consumption among young adults (18 to 25 years old) was 81.9 %, 39.8 % of them consumed alcohol regularly, and the 30-day prevalence of binge drinking (a drinking occasion that includes consumption of at least 60 g of alcohol) was found to be 41.9 % in 2011. With regard to regular alcohol consumption and binge drinking, the share of male participants was almost double to those of their corresponding female counterparts [38]. The number of young people who were to be treated in hospital for their acute alcohol poisoning increased between 2000 and 2008 by 170 % [39].

Meanwhile, it is noteworthy that many studies strongly suggest that alcohol consumption behaviour can be better understood by a social/cultural rather than a biomedical approach [19, 40]. A study among Canadian university students found that in both Caucasian and Chinese ethnic groups, social norms rather than physical symptoms were a significant predictor of alcohol consumption patterns [19]. In a study conducted in the USA, Johnson et al. also reported that China-born Chinese (migrants) consumed much less alcohol compared with America-born Chinese (Americans of Chinese descent) [40].

In recent years, overestimations of peer alcohol use and associations with higher rates of personal use have been demonstrated among European university students [41, 42]. Social norms approach in the case of alcohol consumption may consist of surveying a student population to identify the actual and perceived rates of alcohol consumption, then presenting this information back to the student population through mass media campaigns and a variety of peer education activities [43]. This approach has been found to be an effective method of reducing alcohol intake and alcohol use-related harm on college campuses [1, 44].

According to a European collaborative project aimed at assessing the potential of the social norms approach to reduce alcohol and other drug use among university students (*N* = 4392) in seven countries including Germany, 28 % of male and 49 % of females overestimated the number of drinks per day among their fellow students of the same sex [43]. This suggests that social norms feedback would be useful in correcting the inaccurate perceptions of normative alcohol use in a substantial number of students.

### Limitations of this study

Although a representative sample of students was sought at the universities by selecting courses that represented the different departments/faculties, due to organizational difficulties and variation in response rates, students of health sciences were overrepresented in the final sample in both countries. Therefore, even with the big sample and good response rates, our sample remains a convenience sample, and generalizations of the findings should be made with caution. On the other hand, it was beyond the researchers’ capacity to select a representative sample of university students in two countries, especially for a huge country like China that reveals remarkable economic and developmental differences among regions. Nevertheless, convenience samples are not uncommon in student surveys, e.g. in Europe [45], in the USA [46] or in Australia [47]. As data were self-reported, they may be subject to sources of error, e.g. recall bias and social desirability. Students were recruited during lectures; hence, those who were absent in the class during data collection were not included in the survey. This may have affected the results since absence from lectures might be due to alcohol consumption. Due to the different data collection periods in the two countries, a period effect cannot be excluded. Since different perceived stress scales were applied (PSS-14 in China, PSS-4 in Germany), a direct score comparison of perceived stress between the two countries was not possible. Using regression in separate groups can result in loss of statistical power; moreover, when the sample size for one group is larger than that for the other, it may be possible that a variable shows a significant effect in one group and an insignificant effect in the other, while the difference in effects between the groups may not be actually statistically significant [48]. Since in our study the German subsample is bigger than the Chinese, the statistically significant difference in associations between the two groups should be interpreted in relation to potential methodological limitations. On the other hand, interaction terms may make the odds ratio interpretation more challenging besides the fact that including many interaction terms in the complete sample regression model can also lead to loss of statistical power [48]. Additionally, in the case of a qualitative interaction, i.e. both positive and negative subgroup-specific effects, the overall effect may be close to zero and hence an overall effect may not be demonstrated in the whole sample (in our analyses, the example of the age effect on alcohol consumption frequency—a positive association among the Chinese and a negative association among the Germans; in the whole sample model, no statistical significance between the two variables revealed) [49]. Finally, in a cross-sectional survey, the findings are associations not causations, for instance, it is possible that more drinking is the cause of insufficient income or insufficient income could cause alcohol consumption.

## Conclusions

Despite some limitations, our results provide information for country-specific preventive strategies against drinking in China and Germany. For example, it is important for Chinese universities to provide educational awareness programmes of alcohol use and abuse, since most Chinese students reach the onset age of drinking when they enter university. For the German side, our results imply that alcohol prevention should start before students enter university, as it has been reported that earlier onset age of drinking also predicts heavy drinking in later life [12, 50]. It may be sensible to add the Chinese norm of social network maintenance through having meals rather than drinking together to reduce alcohol consumption among German adolescents and youth in the social norms approach in alcohol reduction. The social norms approach does not put moral pressure on students who decide to use alcohol but rather informs them about the predominant lifestyle choices in their own community. Since the data used in this process are obtained from the students’ own community, it may avoid conflict with the wish for autonomy in decision-making among students but helps them to facilitate ownership [43].

## Abbreviations

95 % CI, 95 % confidence interval; ADH, alcohol dehydrogenase; ALDH, aldehyde dehydrogenase; BMI, body mass index; BZgA, Federal Centre for Health Education; NRW, North Rhine-Westphalia; OR, odds ratio; PKU, Peking University; PSS, Perceived Stress Scale; SYSU, Sun Yat-sen University
